# Action of enfuvirtide on the pregnancy of albino rats ( *Rattus norvegicus albinus, Rodentia, Mammalia* ): biological assay and functional and histological analyses of exposed maternal-fetal organs

**DOI:** 10.31744/einstein_journal/2023AO0230

**Published:** 2023-03-24

**Authors:** Renata Delphim de Moraes, Edward Araujo, Adauto Castelo, Alberto Borges Peixoto, Abês Mahmed Amed

**Affiliations:** 1 Universidade Federal de São Paulo São Paulo SP Brazil Universidade Federal de São Paulo, São Paulo, SP, Brazil.; 2 Universidade de Uberaba Uberaba MG Brazil Universidade de Uberaba, Uberaba, MG, Brazil.

**Keywords:** Rats, Wistar, Pregnancy outcome, Enfuvirtide, Maternal-fetal outcomes, Histological analysis, Biological assay

## Abstract

**Objective:**

To assess the effects of enfuvirtide on pregnancy in albino rats and their fetuses.

**Methods:**

Forty pregnant EPM 1 Wistar rats were randomly allocated into four groups: control (E) (distilled water twice/day), G1 (4mg/kg/day enfuvirtide), G2 (12mg/kg/day enfuvirtide), and G3 (36mg/kg/day enfuvirtide) groups. On the 20th day of gestation, the rats were anesthetized and subjected to cesarean section. Their blood was collected for laboratory analysis, and they were sacrificed. The offspring’s fragments of their kidneys, liver, and placentas and the maternal rats’ fragments of their lungs, kidneys, and liver were separated in the immediate postpartum period for light microscopy analysis.

**Results:**

No maternal deaths occurred. In the second week at the end of pregnancy, the mean weight of the G3 Group was significantly lower than that of the G2 Group (p=0.029 and p=0.028, respectively). Analyzing blood laboratory parameters, the G1 Group had the lowest mean amylase level, and the G2 Group had the lowest mean hemoglobin level and the highest mean platelet count. In the morphological analysis, there were no changes in organs, such as the kidneys and liver, in both the maternal rats and offspring. Three maternal rats in the G3 Group had pulmonary inflammation in the lungs.

**Conclusion:**

Enfuvirtide has no significant adverse effects on pregnancy, conceptual products, or functional alterations in maternal rats.

## Highlights

• No drug related fetal malformations.

• No adverse effects on vital organ functions.

**Figure:**
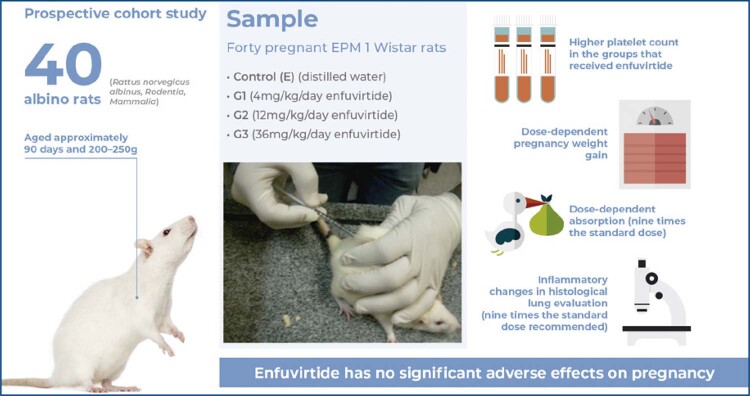


## INTRODUCTION

The rate of mother-to-child transmission of human immunodeficiency virus (HIV) without any intervention in the pregnancy-puerperal cycle is approximately 25.5%. Relatively simple interventions, such as antiretroviral administration, elective cesarean section, peripartum chemoprophylaxis with zidovudine, and contraindication to breastfeeding, decrease HIV transmission from 2% to 0%.^( 1 )^ Antiretroviral therapy has evolved since the beginning of the epidemic, initially with zidovudine monotherapy, dual therapy, and finally highly active antiretroviral therapy, which is most suitable for pregnant women who meet the criteria for starting treatment, aiming to control their infection or reduce vertical HIV transmission. Some factors can prevent the success of suppression and serum viral detection at the time of delivery, such as failure to diagnose the disease, failure to adhere to the proposed treatment, or virological failure (viral resistance to antiretrovirals).^( 2 )^ In the Pediatrics AIDS Clinical Trials Group protocol 076, the development of viral resistance to zidovudine was observed in approximately 2.7% of patients who used this drug alone.^( 3 )^

Despite more than 30 years of therapy and pharmacological development, there remain failures in the response to the recommended therapeutic regimens. Approximately 10-20% of patients cannot achieve optimal serum suppression of the virus for several reasons, including inadequate patient adherence to the drug, presence of comorbidities, and viral resistance.^( 4 , 5 )^ The mutations experienced by HIV, associated with the selective pressure of antiretroviral drugs, can provide HIV “immunity” against antiretroviral drugs. The inappropriate use of medication accelerates the process of selection of resistant viral strains, in addition to cross-resistance to drugs of the same class.^( 6 )^

When there is viral resistance to multiple drugs, the only treatment option for serum viral detection is to modify the drug class to which the virus is resistant. In 2003, a drug from the fusion inhibitor class, enfuvirtide (T-20 Fuzeon®, Roche Laboratory), was approved by the Food and Drug Administration (FDA) to compose therapeutic regimens. This drug is novel in that it prevents the virus from entering the cell. It specifically inhibits viral envelope surface glycoprotein 41, which mediates the fusion of HIV with cluster of differentiation 4 lymphocytes.^( 7 , 8 )^ Enfuvirtide is administered in pregnant women (currently class B according to the FDA), and it should be considered a prophylaxis for mother-to-child transmission of HIV, especially in cases of multidrug-resistant viruses.^( 9 )^ Few studies published until now, mostly case reports, have evaluated the reduction in maternal-fetal transmission of HIV in multidrug-resistant pregnant women using enfuvirtide.^( 10 - 12 )^ Furthermore, the chronic effects of this drug on maternal organs are unknown.

## OBJECTIVE

This study aimed to develop a biological assay using pregnant rats to assess the effect of the chronic use of enfuvirtide on pregnancy outcomes and maternal organs.

## METHODS

This prospective cohort study included 40 albino rats ( *Rattus norvegicus albinus, Rodentia, Mammalia* ) of the EPM 1 Wistar strain aged approximately 90 days and with an initial weight of 200-250g, virgin, from the Central Animal House of the *Escola Paulista de Medicina, Universidade Federal de São Paulo* (EPM-UNIFESP). The animals were kept in collective polyurethane cages measuring 45×30×15cm in length, width, and height, respectively. Initially, four animals were kept in each cage for mating exposure, at a ratio of three females to one male, between 7 pm and 7 am. This study was approved by the Ethics Committee of the UNIFESP (# 1675/06).

Pregnancy was diagnosed using the method of Hamilton and Wolfe:^( 13 )^ the presence of spermatozoa in the mucous material of the vaginal introitus of female rats, collected with cotton swabs, seeded on optical microscope slides, and evaluated at 10× magnification. This day was scored as day zero of the pregnancy. After pregnancy diagnosis, the rats were kept in cages, three animals in each cage (30cm×15cm×12cm), with standardized food (Nuvital CR-1) and acidified water *ad libitum* . The artificial lighting used was a 40-watt fluorescent lamp (Phillips brand, daylight model) in a photo period of 12-hours light and 12-hours dark, considering the light period between 7 am and 7 pm and dark period between 7 pm and 7 am.

The animals were randomly divided into four study groups: control (E) (stress), G1, G2, and G3 Groups. The animals in the G1, G2, and G3 Groups were subcutaneously administered a standardized substance (dose dependent on each Group) in the dorsal region or cervical fold using sterile and disposable material Portex^®^ Hypodermic Needle-pro™ (Smiths Medical, Inc.) (Syringe & Needle with Needle Protection Device), containing a 27 G × ^½^” needle with a protective cover attached to the 1mL/Ls syringe. The drug used was enfuvirtide (T20 Fuzeon^®^, Roche Laboratory), which was supplied by the Brazilian Ministry of Health (batch 3581462 TE-BR 0001). Each vial contained enfuvirtide, presented as a white, sterile powder, lyophilized for subcutaneous injection after reconstitution with the diluent, and supplied in a separate vial. After reconstitution with 1.1mL of diluent (sterile water), the solution contained 90mg/mL of enfuvirtide, standardized for use in humans weighing ≥60kg.

The control (E) (stress) was the Control Group that comprised 10 animals who were subcutaneously injected with 0.05mL distilled water (medicated vehicle), from 12/12 hours, between 7 am and 7 pm, from the 1st to the 20th day of pregnancy. The dose (0.05mL) was equivalent to the volume of medication administered to the G1 Group. The G1 Group was an experimental group comprising 10 animals, which received 0.5mg of enfuvirtide, 2.0mg/kg (standard dose recommended for humans), with a base weight of 250g for each rat. The dose was diluted in 0.05mL distilled water, as a subcutaneous injection, every 12 hours, between 7 am and 7 pm, from the 1st to the 20th day of pregnancy. The total daily dose was 4mg/kg. The G2 Group was an experimental group comprising 10 animals, which received 1.5mg of enfuvirtide, 6.0mg/kg (three times the standard dose recommended in humans), with a base weight of 250g for of each rat. The dose was diluted in 0.05mL distilled water, as a subcutaneous injection, every 12/12 hours, between 7 am and 7 pm, from the 1st to the 20th day of pregnancy. The total daily dose was 12mg/kg. The G3 Group was an experimental group comprising 10 animals, which received 4.5mg of enfuvirtide, 18mg/kg (nine times the standard dose recommended for humans), with a base weight of 250g for each rat. The dose was diluted in 0.05mL distilled water, as a subcutaneous injection, between 7 am and 7 pm from the 1st to the 20th day of pregnancy. The total daily dose was 36mg/kg.

During the gestational period, the weight of the rats in each group was measured on days 0, 7, 14, and 20 of pregnancy using a precision digital semi-analytical scale model AM5500 (Marte Científica, Santa Rita do Sapucaí, Brazil). On the 20th day of gestation, the animals were subjected to general anesthesia with 20mg/kg xylazine and 100mg/kg ketamine via intra-abdominal injection.

Once anesthesia was completed, the rats were dissected through a longitudinal, median, sterno-pubic incision using a 24-inch blade and scalpel handle, thus exposing their internal organs. In total, 5-7mL of intracardiac maternal blood was collected by direct puncture (ventricular cavity) for laboratory serum analysis using a 10mL sterile syringe and 25 × 7 22-G needle, gently and continuously, taking to the exsanguination of the anesthetized animal, until the heartbeat stops. The blood collected from each rat was divided equally into a test tube with ethylenediaminetetraacetic acid and another dry tube provided by the laboratory and then sent for laboratory processing. The following examinations were performed: complete blood count and blood glucose, total cholesterol, triglyceride, creatinine, urea, amylase, hepatic transaminase (glutamic-oxaloacetic transaminase and glutamic pyruvic transaminase), and creatine kinase MB (CKMB) level assessment.

Subsequently, a cold scalpel hysterotomy was performed immediately to remove the (live) conceptuses and analyze the offspring: number of offspring, total weight of offspring, number of placentas, total weight of placentas, number of resorptions, number of fetal deaths, and diagnosis of major malformations (such as neural tube defects, craniofacial malformations, limb malformations, abdominal wall defects - each animal was analyzed macroscopically using a magnifying glass). The offspring were then subjected to immediate cervical dislocation. Kidney and liver samples from each litter were prepared for histological analysis from all fetuses, except for one of them separately in each group, for the same study of the entire conceptus. Subsequently, fragments of the kidney, liver, lung, and popliteus muscle were removed from each adult animal for morphological analysis of these organs and structures and sent for a complementary study in a histological laboratory.

The biological material was stored in a sterile plastic container with a 60mL sealing cap filled with a buffered formaldehyde solution (phosphate buffer) for a period of 24 hours, with the solution being changed every 12 hours. Subsequently, they were dehydrated in absolute alcohol, with the clean solution changed every hour for 3 hours. After dehydration, the biological material was diaphonized in xylene in two consecutive baths, lasting 1 hour each. After the baths, they were impregnated with liquid paraffin and kept in a heated oven at 60-70°C for 4-6 hours. Finally, the cells were blocked and identified. The blocks were cut using a LEICA RM 2145 microtome (Ramsey, MN, USA), which was adjusted to a 5mm thickness. The sections were prepared on slides previously smeared with Mayer’s albumin and kept in ovens at 37°C for 24 hours for drying and gluing. Hematoxylin and eosin staining was then performed. The slides were examined under a Zeiss Axiostar Plus microscope (Oberkochen, Germany).

Data were transferred to an Excel 2019 spreadsheet (Microsoft Corp., Redmond, WA, USA) and analyzed using the Statistical Package for the Social Sciences version 20.0 (IBM Corporation, Armonk, NY, USA) and Prism version 7.0 (GraphPad Software, San Diego, CA, USA). Quantitative variables were analyzed using a normality test (D’Agostino-Pearson), and those with a normal distribution are presented as means and standard deviations. Variables with non-normal distributions are presented as medians and minimum and maximum values. Categorical variables were described based on absolute and percentage frequencies and are presented in the tables. To study the differences between categorical variables and their proportions, the χ^2^ test was used. Analysis of variance (ANOVA) was used for normally distributed variables to study the differences between continuous variables, with one-factor and Tukey’s multiple comparison method. The Kruskal-Wallis test was used for non-normally distributed variables. Dunn’s post hoc test was used for pairwise comparisons. ANOVA with repeated measurements was used to compare repeated measurements of animals’ weight in different periods of this study. The numbers of absorptions, malformations, and deaths were analyzed using Fisher’s exact test. The significance level for all tests was set at α<0.05.

The GPower version 3.1 software (Heinrich-Heine- Universität, Düsseldorf, Germany) was used to calculate the post hoc power of this study. The post hoc power of this study to analyze the effect of chronic use of enfuvirtide on continuous variables, based on a *w* effect of 0.50, an α error probability of 0.05, and a total sample size of 40 subjects in the four groups, was 70.9%. The post hoc power of this study to analyze the effect of the chronic use of enfuvirtide on paired continuous variables, based on a *w* effect of 0.50, an α error probability of 0.05, a total sample size of 40 subjects in the four groups and four repeated measurements, was 90%. The post hoc power of this study to analyze the association between chronic use of enfuvirtide and categorical variables, based on an α error probability of 0.05 and a noncentrality parameter of 10, with two degrees of freedom was 81%.

## RESULTS

**Figure 1:**
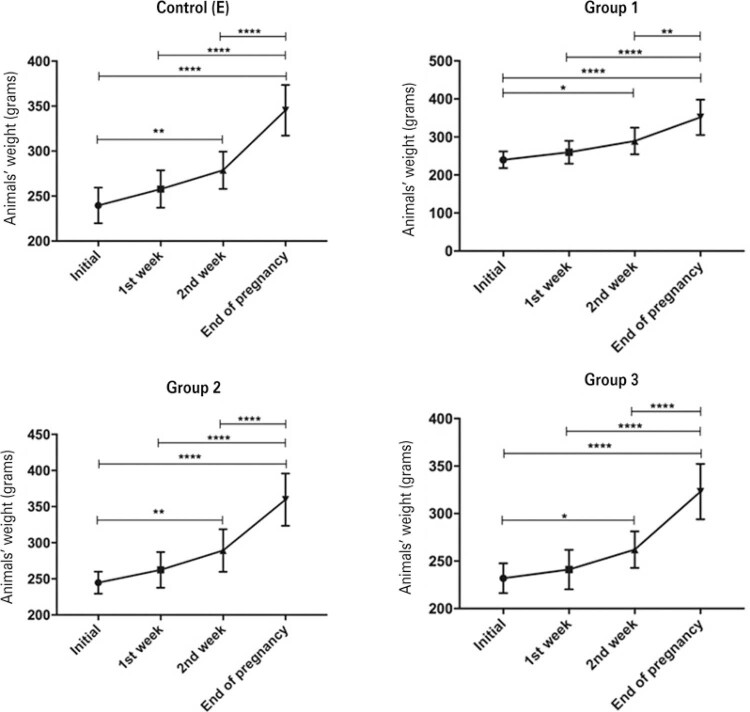
Effect of the chronic use of enfuvirtide on the weight gain of the control (E) (p<0.0001), G1 (p<0.0001), G2 (p<0.0001), and G3 (p<0.0001) groups

The mean weight of the maternal rats was measured at four time points during pregnancy: at the beginning, at 1 week, at 2 weeks, and at delivery. There was a significant effect of the chronic use of enfuvirtide on the mean weight gain of the control (E) (p<0.0001), G1 (p<0.0001), G2 (p<0.0001), and G3 (p<0.0001) groups during the four time points of weight measurement during pregnancy ( Figure 1 ).

Although the G3 Group had a lower mean weight at the four time periods than the other groups, there was no statistically significant difference between this group and the control (E). The main difference found was between the G3 and G2 Groups; in the second week and at delivery, the mean weight of the G3 Group was significantly lower than that of the G2 Group (p=0.029 and p=0.028, respectively).

Analyzing the number of offspring, the only treated Group that differed from the control (E) was the G2 Group (p=0.005), which produced a greater number of offspring (in total, there were 126 *versus* 100 in the control (E). In the G2 Group, the minimum number of offspring conceived was seven, and half of the rats had 12 offspring. The mean weight of the offspring was also slightly higher in the G2 Group than in the other groups, with no statistical difference in relation to the control (E) (p=0.507). The G1 and G3 Groups also did not differ significantly from the control (E) (p=0.777 and p=0.233, respectively). The mean weight of the offspring in the G3 Group was statistically lower than that in G1 (p=0.032) and G2 (p=0.010) groups. The mean placental weight was higher in the G2 and G3 Groups than in the control (E) ( Table 1 ).

**Table 1:** Comparison among groups: number of offspring and mean weight of offspring and placentas

	Control (E)	G1	G2	G3	p value^†^
Number of offspring/placentas	10.5	11.5	12.0*	11.0	0.024
Median (IIQ)	(9.5-11)	(9-13.5)	(12-14)	(8.8-11.5)	
Mean weight of offspring (grams)	4.1	4.3^§^	4.4^§^	3.7	0.010
Median (SD)	(0.6)	(0.4)	(0.6)	(0.2)	
Mean weight of placentas (grams)	0.62	0.70	0.78*	0.79*	0.008
Median (SD)	(0.11)	(0.14)	(0.07)	(0.14)	

^†^ Concerning the hypothesis of equality among the four groups: Kruskal-Wallis test for number of offspring and analysis of variance for mean weights; ^§^ p<0.05 when compared to the G3 Group; * p<0.05 when compared to the control (E).

In the G3 Group, four maternal rats had absorption (two rats with one, one with two, and one with three), denoting a greater number of dose-dependent absorption (p=0.072). In the control (E), one maternal rat had an absorption. There were no absorption bands in the G1 and G2 Groups. There was only one malformation, which occurred in the G2 Group. Two deaths were observed, one in the control (E) and one in the G2 Group. No differences were observed among the Groups in terms of malformation and mortality ( Table 2 ).

**Table 2:** Comparison among groups: absorptions, malformations, and mortalities

	Control (E)	G1	G2	G3	p value^†^
Number of absorptions					
None	9	10	10	6	0.072*
1	1	-	-	2	
2	-	-	-	1	
3	-	-	-	1	
Malformations	-	-	1	-	>0.999
Mortalities	1	-	1	-	>0.999

^†^ Concerning the hypothesis of equality among the four groups by Fisher’s exact test; * G1 and G2 Groups *versus* control (E) (p>0.999), G3 Group *versus* control (E) Group (p=0.303), and G1 and G2 Groups *versus* G3 Group (p=0.087).

The G2 Group had the lowest mean hemoglobin level and the highest mean platelet count, and the G3 Group had the lowest mean basophil count. Among the treatment groups, the G1 Group had a lower mean CKMB level than the G2 (p=0.033) and G3 (p=0.023) Groups ( Table 3 ).

**Table 3:** Comparison among groups: laboratory parameters

Variables	Groups				p value^†^

	Control (E)	G1	G2	G3	
Hemoglobin (g/dL)	11.5	11.4	10.7^*^	11.4	0.028
Mean (SD)	(0.5)	(0.7)	(0.4)	(1.0)	
Hematocrit (%)	30.4	30.2	28.8	31.5	0.114
Mean (SD)	(1.6)	(1.9)	(1.2)	(3.8)	
Hematocrit (%)	7,795	7,560	6,970	7,210	0.909
Mean (SD)	(2651.9)	(3780.7)	(2677.1)	(1129.9)	
Eosinophils (cel./mm^3^)	1.5	2	1	2	0.226
Mean (SD)	(1-2)	(1-4)	(0.8-2)	(0-2.3)	
Basophils (cel./mm^3^)	1.5	0.5	1	0*	0.138
Mean (SD)	(0-4.5)	(0-1)	(0-3)	(0-1)	
Platelets (number/µL)	766,700	812,100	895,800^*^	809,500	0.014
Mean (SD)	(43673.2)	(85345.6)	(120939.7)	(55616.2)	
Urea (mg/dL)	57.2	52.8	52.9	61.5	0.050
Mean (SD)	(4.9)	(5.6)	(8.1)	(10.9)	
GOT (UI/L)	88.7	88.8	98.9	106.1	0.498
Mean (SD)	(27.8)	(41.3)	(25.3)	(20.8)	
GPT (UI/L)	65.0	65.7	61.0	69.7	0.167
Mean (SD)	(6.4)	(11.1)	(6.2)	(9.0)	
Amylase (UI/L)	1228,2	1161,2*	1207,3	1217,4	0.800
Mean (SD)	(36.9)	(47.5)	(34.0)	(52.1)	
Glucose (mg/dL)	117.4	110.2	104.1	115.1	0.161
Mean (SD)	(13.0)	(11.4)	(18.3)	(11.4)	
CKMB (ng/mL)	571.4	392.7	724.3^§^	579.8^§^	0.048
Mean (SD)	(352.9)	(138.1)	(287.6)	(112.9)	
Cholesterol (mg/dL)	106.6	94.7	101.4	105.2	0.181
Mean (SD)	(12.8)	(12.2)	(14.3)	(12.1)	
Triglycerides (mg/dL)	495.0	386.6	356.5	464.2	0.387
Mean (SD)	(242.7)	(184.0)	(196.2)	(174.1)	

^†^ Concerning the hypothesis of equality among the four groups: Kruskal-Wallis test for eosinophils and basophils; analysis of variance for the other variables; * p<0.05 when compared to the control (E); § p<0.05 when compared to the G1 Group: G2 (p=0.033) and G3 (p=0.023).

**Figure 2:**
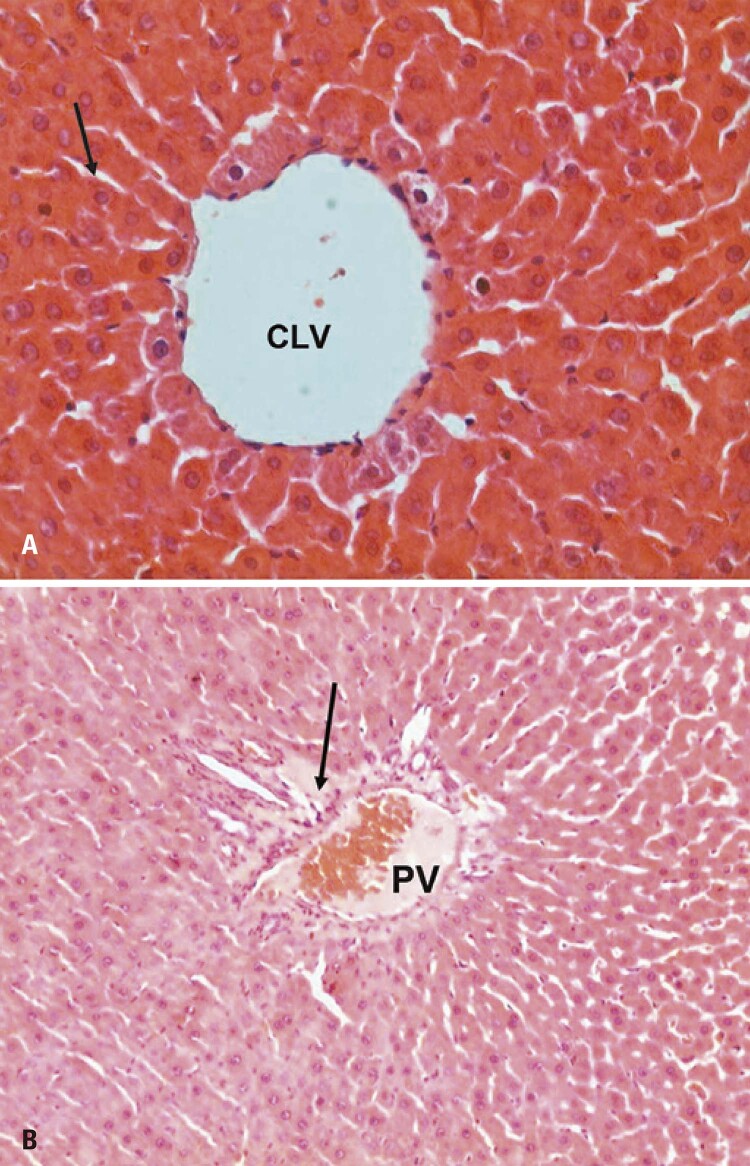
Hematoxylin and eosin staining. Photomicrograph of the maternal hepatic lobe of the control (E). (A) Note the centrilobular vein (CLV) and cords of hepatocytes interspersed with sinusoid capillaries (arrows) and the normal appearance of hepatocytes; (B) Focal congestion of the zone III periportal space (arrow)

The control (E), G1, G2, and G3 Groups were similar in morphological evaluation of the maternal liver by light microscopy. According to hematoxylin and eosin staining results, the liver was well preserved and homogeneous, and the hepatic lobules, portal spaces, and hepatic veins were easily identified. The liver parenchyma consisted of hepatocytes arranged in the form of a cord (or Remak’s plaques), converging into the centrilobular vein. Hepatocytes were voluminous polyhedral cells, mono- or binucleate, with central spherical nuclei rich in euchromatin, with evident nucleolus. The cytoplasm contained basophilic and eosinophilic areas, rendering it heterogeneous. Hepatic sinusoids were observed between hepatocytes. The histology of the portal space was preserved, with mild dilatation of the portal vein and congestion of the periportal space in zone III, a common finding due to adaptation to pregnancy ( Figure 2 ).

**Figure 3:**
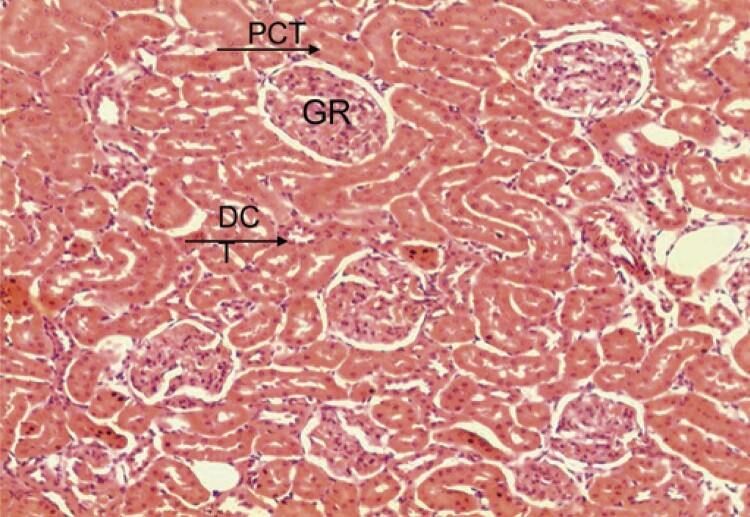
Hematoxylin and eosin staining. Photomicrograph of the cortical portion of the maternal kidney of the control (E). Note the typical renal glomerulus, proximal convoluted tubules (PCT), and some distal convoluted tubules (DCT)

The control (E), G1, G2, and G3 Groups were similar in the morphological evaluation of the maternal kidney by light microscopy. According to hematoxylin and eosin staining results, the kidney was well preserved, and the renal corpuscles and proximal and distal convoluted tubules were identified. The glomeruli were composed of capillaries, podocytes, and endothelial and mesangial cells. The capsular space and Bowman’s capsule were also present. In the cortical region, the renal parenchyma consisted of proximal tubules, formed by cubic or polyhedral cells, with eosinophilic cytoplasm and a rounded basal nucleus. The striated border was clearly visible at the apex of the cells. In the renal cortical portion, distal convoluted tubules with evident light were formed by cubic cells with large nuclei. In the medullary portion of the kidney, loops of Henle (thick and thin segments) were identified along the capillaries and collecting tubules. The thin segment of the loops of Henle was difficult to distinguish because of its resemblance to blood capillaries. The collecting tubules were composed of cubic cells, well-defined cytoplasm, and spherical nucleus ( Figure 3 ).

**Figure 4:**
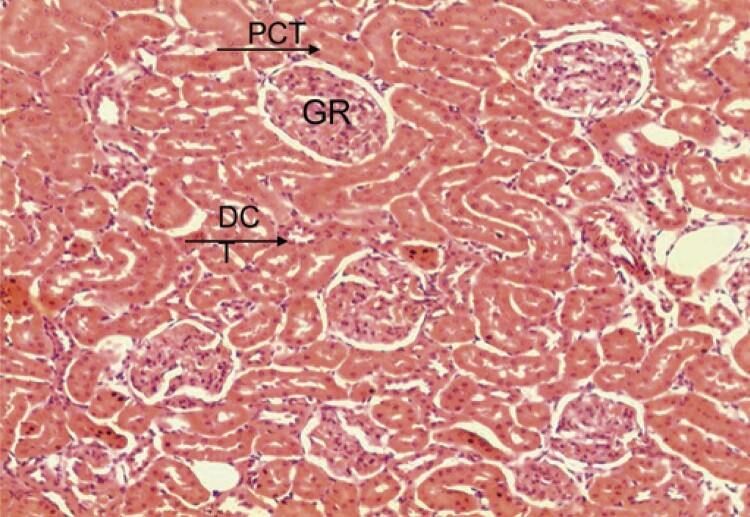
Hematoxylin and eosin staining. Photomicrograph of the bronchiolar and alveolar portions of the maternal lungs of the control (E). Note the bronchioles (broc), alveoli (alv), and proper lymphoid tissue (LT)

The control (E), G1, and G2 Groups were similar in the morphological evaluation of the maternal lung by light microscopy. According to hematoxylin and eosin staining results, the bronchi were composed of simple ciliated cylindrical epithelium, a smooth muscle layer, and mucous or mixed glands, whose ducts opened into the bronchial lumen. In the adventitial layer (connective tissue layer surrounding the cartilaginous parts) and mucosa, we observed the accumulation of lymphocytes and lymph nodes, mainly at the branching points of the bronchial tree. The bronchioles had no cartilage, glands, or lymph nodes. The initial epithelium was of the ciliated simple cylindrical type and became a simple cubic in the final portion. After the mucosa, there was a smooth muscle layer, whose cells were intertwined with elastic fibers. The respiratory bronchiole was lined by a simple epithelium, which varied from low columnar to cuboidal. On the wall, we observed alveoli and small sac-shaped evaginations, which had three distinct cell types: capillary endothelial cells, type I pneumocytes, and type II pneumocytes. The alveolar walls were coated with a thin surfactant film ( Figure 4 ).

**Figure 5:**
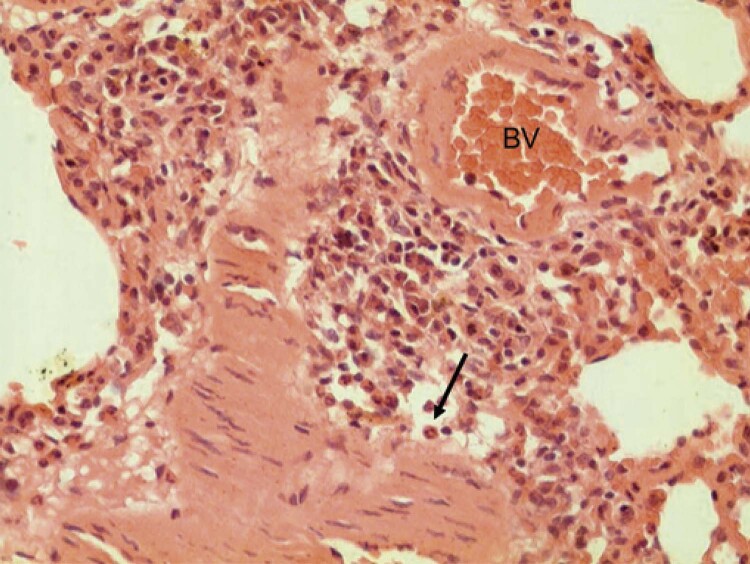
Hematoxylin and eosin staining. Photomicrograph of the bronchiolar and alveolar portions of the lung of maternal rats of the G3 Group (number 9). Note the intra-alveolar polymorphonuclear (arrows), congested blood vessels (BV)

Figure 5 shows the maternal lungs of the G3 Group (rats 8, 9, and 10). According to hematoxylin and eosin staining results, the bronchi were composed of simple ciliated cylindrical epithelium, a smooth muscle layer, and mucous or mixed glands, whose ducts opened into the bronchial lumen. In the adventitial layer (connective tissue layer surrounding the cartilaginous parts) and mucosa, we observed the accumulation of lymphocytes and lymph nodes, mainly at the branching points of the bronchial tree. The bronchioles had no cartilage, glands, or lymph nodes. The initial epithelium was of the ciliated simple cylindrical type and became a simple cubic in the final portion. After the mucosa, there was a smooth muscle layer, whose cells were intertwined with elastic fibers. The respiratory bronchiole was lined by a simple epithelium, which varied from low columnar to cuboidal. On the wall, we observed alveoli and small sac-shaped evaginations. In this group, polymorphonuclear cells and intra-alveolar neutrophils were observed, which was a probable sign of local inflammation. Notably, local vascular congestion and polymorphonuclear cells exited the blood vessels.

The morphological evaluation of the fetuses was similar in all groups when the kidney, liver, and lungs were evaluated, with no abnormal findings.

## DISCUSSION

The present study in maternal rats suggests that the use of enfuvirtide during pregnancy, even at doses up to nine times higher than those recommended for humans, does not interfere with obstetric or neonatal outcomes. The use of the drug during the animals’ entire pregnancy allowed the observation of the effects (dose-dependent) of enfuvirtide on the number and weight of offspring, risk of miscarriage, intrauterine death, congenital malformations, and laboratory changes in the maternal rats.

In cases of multidrug-resistant pregnant women, due to low HIV therapy adherence or viral genetic factors, the use of new classes or drug associations is imperative to reduce maternal-fetal transmission rates. In these situations, the use of enfuvirtide can be beneficial because it is an injectable drug that favors adherence and has good tolerance, quick efficacy, and no placental transfer. In a small series of seven cases that evaluated the maternal-fetal outcomes of pregnant women receiving enfuvirtide, the average use of the drug was 30 days. All newborns were seronegative, without congenital anomalies, and umbilical cord dosages were negative, with six delivered by cesarean section and one by vaginal delivery.^( 14 )^

Reis et al.^( 15 )^ evaluated the resistance of 124 pregnant Brazilian women to enfuvirtide. The natural resistance rate to enfuvirtide was 6.1%, and 20.4% had compensatory mutations in HR2. They concluded that natural resistance to enfuvirtide was not associated with *pol* resistance or previous use of antiretroviral drugs. The high rate of secondary resistance, including multidrug resistance, indicates that the number of pregnant women who may need enfuvirtide salvage therapy may be higher than anticipated.

In a study using an *ex vivo* human placental perfusion model, Ceccaldi et al.^( 16 )^ did not observe placental transfer of enfuvirtide, even at maternal concentrations that were twice the therapeutic levels. The molecular weight of the molecule (4492 kDa) and its ionized state partially or completely prevent vertical placental transfer, suggesting that the drug can be used in HIV-infected pregnant women without causing fetal exposure. In our study, the mean weight of the offspring in the groups that received enfuvirtide was similar to that of the control (E). Similarly, only one congenital malformation (amniotic band sequence) was detected in the group that received three times the recommended dose for humans. This change is unlikely to be attributable to exposure to enfuvirtide, as this drug does not cross the placenta. Enfuvirtide does not appear to penetrate the genital tract, and placental transfer is negligible, indicating that its primary mode of action is, therefore, likely to be via a reduction in maternal plasma viremia.^( 17 )^

Shust et al.^( 18 )^ assessed pregnant women with multidrug resistance, in which salvage regimens containing several drugs, such as darunavir, etravirine, raltegravir, or enfuvirtide, were provided for adequate maternal-child transmission control of HIV. All pregnant women were anemic in the second trimester. None of the patients had elevated transaminase or bilirubin levels or required discontinuation of salvage regimens owing to toxicity or intolerance. Preterm deliveries occurred in two cases, with 7/8 by cesarean sections and one by vaginal delivery. Four of the eight newborns were small for gestational age. One newborn died 9 h after delivery because of prematurity. Appropriate neurological and motor milestones were observed in all the children at 6 months of age. In our experimental study, we observed anemia in maternal rats of the G2 Group. We also observed higher platelet count in the groups that received enfuvirtide compared with that in the control (E). Creatine kinase MB levels decreased in the G1 Group and increased in the G3 Group.

In the histological evaluation of the lungs of the maternal rats in the control (E), G1, and G2 Groups, there was no perceptible toxic effect on the referred viscera, without detection of notable morphological alterations under light microscopy. In the G3 Group, which received nine times the dose recommended by enfuvirtide for humans, we observed inflammatory changes, such as the presence of polymorphonuclear cells and intra-alveolar neutrophils, local vascular congestion, and polymorphonuclear cells coming out of the blood vessels, which are probable signs of local inflammation. No human studies have evaluated the microscopic effect of enfuvirtide on the maternal lung. Clinicians should be aware of the possible side effects of this drug during pregnancy.

This study has potential limitations. The findings involving offspring’s numbers comparison, specifically between G3 and G2 must be seen considering some limitation. The sample bias results due to rats randomized allocation in groups, unintentionally grouped slightly lower weighed rats in G3. This consideration may impact the number of offspring in this specific group. The bias does not affect the present results evolving drug adverse effects.

## CONCLUSION

In summary, the present study did not find any significant adverse effects of enfuvirtide on pregnancy, conceptual products, or functional alterations in maternal rats, except in the maternal lung, which showed probable signs of local inflammation.

## References

[1] Cooper ER, Charurat M, Mofenson L, Hanson IC, Pitt J, Diaz C, Hayani K, Handelsman E, Smeriglio V, Hoff R, Blattner W, Women and Infants’ Transmission Study Group (2002). Combination antiretroviral strategies for the treatment of pregnant HIV-1-infected women and prevention of perinatal HIV-1 transmission. J Acquir Immune Defic Syndr.

[2] Gallant JE (2007). Approach to the treatment-experienced patient. Infect Dis Clin North Am.

[3] United Nations Programme on HIV/AIDS (UNAIDS) (c2022). AIDS epidemic up-to-date. A global view of HIV infection: 2006b Global Report prevalence map. Source: 2006 Report on the global AID 2006.

[4] McCluskey SM, Siedner MJ, Marconi VC (2019). Management of Virologic Failure and HIV Drug Resistance. Infect Dis Clin North Am.

[5] Karade S, Chaturbhuj DN, Sen S, Joshi RK, Kulkarni SS, Shankar S (2018). HIV drug resistance following a decade of the free antiretroviral therapy programme in India: a review. Int J Infect Dis.

[6] Barbosa MT, Struchiner CJ (2003). Impact of antiretroviral therapy on the magnitude of the HIV/AIDS epidemic in Brazil: various scenarios. Cad Saude Publica.

[7] Bienvenu B, Krivine A, Rollot F, Pietri MP, Lebault V, Meritet JF (2006). A cohort study of enfuvirtide immunological and virological efficacy in clinical practice. J Med Virol.

[8] Mandelbrot L, Landreau-Mascaro A, Rekacewicz C, Berrebi A, Bénifla JL, Burgard M, Lachassine E, Barret B, Chaix ML, Bongain A, Ciraru-Vigneron N, Crenn-Hébert C, Delfraissy JF, Rouzioux C, Mayaux MJ, Blanche S, Agence Nationale de Recherches sur le SIDA (ANRS) (2001). Lamivudine-zidovudine combination for prevention of maternal-infant transmission of HIV-1. JAMA.

[9] Cohan D, Feakins C, Wara D, Petru A, McNicholl I, Schillinger D (2005). Perinatal transmission of multidrug-resistant HIV-1 despite viral suppression on an enfuvirtide-based treatment regimen. AIDS.

[10] Sued O, Lattner J, Gun A, Patterson P, Abusamra L, Cesar C (2008). Use of darunavir and enfuvirtide in a pregnant woman. Int J STD AIDS.

[11] Furco A, Gosrani B, Nicholas S, Williams A, Braithwaite W, Pozniak A (2009). Successful use of darunavir, etravirine, enfuvirtide and tenofovir/emtricitabine in pregnant woman with multiclass HIV resistance. AIDS.

[12] Madeddu G, Calia GM, Campus ML, Lovigu C, Mannazzu M, Olmeo P (2008). Successful prevention of multidrug resistant HIV mother-to-child transmission with enfuvirtide use in late pregnancy. Int J STD AIDS.

[13] Hamilton JB, Wolfe JM (1938). The effect of male hormone substance upon birth and prenatal development in the rat. Anat Rec.

[14] Jeantils V, Alloui C, Rodrigues A, Bentata M, Peytavin G, Carbillon L (2009). Use of enfurvitide in pregnancy in HIV positive women in seven cases. Gynecol Obstet Fertil.

[15] Reis MN, Alcântara KC, Cardoso LP, Stefani MM (2014). Polymorphisms in the HIV-1 gp41 env gene, natural resistance to enfuvirtide (T-20) and pol resistance among pregnant Brazilian women. J Med Virol.

[16] Ceccaldi PF, Ferreira C, Gavard L, Gil S, Peytavin G, Mandelbrot L (2008). Placental transfer of enfuvirtide in the ex vivo human placenta perfusion model. Am J Obstet Gynecol.

[17] Brennan-Benson P, Pakianathan M, Rice P, Bonora S, Chakraborty R, Sharland M (2006). Enfurvitide prevents vertical transmission of multidrug-resistant HIV-1 in pregnancy but does not cross the placenta. AIDS.

[18] Shust GF, Jao J, Rodriguez-Caprio G, Posada R, Chen KT, Averitt A (2014). Salvage Regimens Containing Darunavir, Etravirine, Raltegravir, or Enfuvirtide in Highly Treatment-Experienced Perinatally Infected Pregnant Women. J Pediatric Infect Dis Soc.

